# Maternal Low-Dose Lipopolysaccharide Exposure and House Dust Mite Challenge Alter Serum IgG N-Glycosylation in Offspring Mice

**DOI:** 10.3390/biomedicines14091942

**Published:** 2026-08-29

**Authors:** Ran Zhao, Xiuli Gong, Na Dong, Xiaoyan Dong

**Affiliations:** 1 Department of Respiratory, Shanghai Children’s Hospital, School of Medicine, Shanghai Jiao Tong University, Shanghai 200000, China; 2 NHC Key Laboratory of Medical Embryogenesis and Developmental Molecular Biology & Shanghai Key Laboratory of Embryo and Reproduction Engineering, Shanghai 200000, China; 3 Shanghai Institute of Medical Genetics, Shanghai Children’s Hospital, School of Medicine, Shanghai Jiao Tong University, Shanghai 200000, China; 4 Institute of Pediatric Infection, Immunity, and Critical Care Medicine, School of Medicine, Shanghai Jiao Tong University, Shanghai 200000, China

**Keywords:** IgG, N-glycan, maternal treatment, lung inflammation, asthma

## Abstract

**Background**: The “hygiene hypothesis” and “farm effect” suggest that early-life microbial exposure protects against allergic diseases. Maternal low-dose lipopolysaccharide (LPS) exposure during pregnancy attenuates allergic airway inflammation in offspring, yet the underlying mechanisms remain incompletely understood. Immunoglobulin G (IgG) N-glycosylation critically regulates antibody effector functions and maternal–fetal immune transfer, but its role in transgenerational allergy protection is unknown. **Objective:** To investigate whether maternal low-dose LPS exposure modulates serum IgG N-glycan profiles in offspring and whether these glycomic changes are associated with protection against house dust mite (HDM)-induced allergic airway inflammation. **Methods:** Pregnant C57BL/6J mice received intraperitoneal LPS (7 µg/kg) or phosphate-buffered saline (PBS) on gestational day 15. Offspring were sensitized with HDM extract or PBS at 6 weeks of age. Serum IgG was purified using Protein G affinity chromatography, and N-glycans were released with PNGase F, labeled with 2-aminobenzamide, and analyzed by ultra-performance liquid chromatography (UPLC) with fluorescence detection. Twelve glycan peaks (GP1-GP12) and five derived traits (galactosylation, sialylation, monogalactosylation, digalactosylation, and bisecting glycans) were quantified. **Results:** HDM challenge significantly altered offspring IgG glycosylation, characterized by decreased digalactosylation, total galactosylation, and sialylation, consistent with inflammatory glycan signatures reported in other inflammatory diseases. Maternal LPS exposure alone induced similar glycomic shifts in offspring (decreased digalactosylation, total galactosylation, and sialylation). In HDM-challenged offspring, maternal LPS pretreatment produced a trend toward reduced lung inflammation without statistical significance; however, IgG glycan differences between LPS-HDM and PBS-HDM groups were non-significant. **Conclusions:** Maternal low-dose LPS exposure reduced offspring IgG galactosylation and sialylation. HDM challenge produced similar glycomic changes. Despite a trend toward attenuation of HDM-induced lung inflammation, maternal LPS did not restore IgG glycosylation. These findings provide first evidence linking prenatal immune stimulation to transgenerational IgG glycomic remodeling.

## 1. Introduction

Allergic asthma is one of the most common chronic airway diseases worldwide, with its prevalence continuing to rise and placing a substantial burden on public health systems [1]. The “hygiene hypothesis” and the “farm effect” suggest that abundant microbial exposure during early life may reduce the risk of allergic diseases through immunomodulatory mechanisms [2,3]. Many animal studies have demonstrated that maternal low-dose lipopolysaccharide (LPS) or bacterial lysate exposure during pregnancy attenuates allergic airway inflammation in offspring [4,5,6,7]. However, the underlying molecular mechanisms remain incompletely understood.

IgG is the only antibody isotype that can cross the placenta [8], and it plays a key role in maternal–fetal immune transfer and in the development and function of the offspring immune system after birth [9,10]. Studying the features of maternal IgG can therefore provide useful information for understanding transgenerational immune regulation. N-glycosylation is the most common and functionally important post-translational modification of IgG. It regulates antibody binding to Fcγ receptors and complement C1q, and it strongly affects antibody effector functions such as antibody-dependent cellular cytotoxicity, complement-dependent cytotoxicity, and inflammatory modulation [11,12]. Previous studies have shown that galactosylation and sialylation of IgG N-glycans are reduced in several chronic inflammatory diseases (including systemic lupus erythematosus and inflammatory bowel disease), in cancer, and with aging [13]. These glycomic changes are useful biomarkers for distinguishing disease from healthy states. IgG N-glycosylation patterns also affect placental transport efficiency [14] and the initiation and regulation of immune responses in offspring. However, systematic studies on the effects of maternal low-dose LPS exposure on offspring IgG N-glycosylation profiles are still limited.

This study established four experimental groups: PBS-PBS (control), PBS-HDM (HDM sensitization), LPS-PBS (maternal LPS exposure), and LPS-HDM (maternal LPS plus offspring HDM challenge). Through these comparisons, we aimed to investigate the transgenerational effects of prenatal LPS exposure on offspring IgG glycomics and to provide glycoimmunological evidence for understanding allergic airway inflammation.

## 2. Materials and Methods

### 2.1. Experimental Animals, Feed and Environment

Six-week-old C57BL/6J mice (Shanghai SLAC Laboratory Animal Co. Shanghai, China) were raised at the Animal Experiment Facility of the Shanghai Institute of Medical Genetics, Children’s Hospital, affiliated with Shanghai Jiao Tong University. The temperature was 22 °C, the humidity was 50%~70%, and a 12 h automatic day and night cycle was maintained; water and feed were provided ad libitum. All the mice were acclimated to the environment for 1 week before the intervention. All animal experimental procedures were performed in strict accordance with the Guide for the Care and Use of Laboratory Animals. All procedures were approved by the Animal Care and Use Committee of Shanghai Children’s Hospital (SHCH-IACUC-2024-XMSB-125, 18 September 2024).

### 2.2. Animal Grouping and Model Establishment

We designed the four mouse groups to address three questions: whether HDM-induced allergic airway inflammation alters serum IgG N-glycosylation profiles in offspring, whether maternal low-dose LPS exposure alone is sufficient to alter offspring IgG glycosylation, and whether maternal LPS pretreatment modulates offspring IgG glycosylation under HDM sensitization. The pregnant mice were treated with LPS and phosphate-buffered solution (PBS), and the progeny were treated with HDMs or PBS at 6 weeks. There were 6 mice in each group: PBS-PBS, PBS-HDM, LPS-PBS and LPS-HDM. Only male offspring were included to minimize sex-related variability and estrous cycle-associated hormonal confounding. LPS was intraperitoneally injected at 15th days of gestation at a dose of 7 µg/kg, which has been verified as a safe and effective dose, and not to cause premature birth or miscarriage during pregnancy [15], and the mice in the control group were injected with an equal volume of PBS. The mice in the HDM group were sensitized by intraperitoneal injection of 80 μL (100 μg) of HDM extract on day 1, 8 and 15 of the experiment and were given 20 μL (10 μg) of HDM nasal drops in the same nasal cavity from day 16 to day 20. The mice in the control group were injected with an equal volume of PBS and given a constant volume of PBS via nasal drops. Offspring were sacrificed on day 21 following HDM or PBS treatment, and serum was harvested.

### 2.3. Hematoxylin–Eosin (HE) Staining and Scores

Pathological changes in the lung tissues of the mice were assessed by HE staining, performed as previously described [16]. Inflammation was graded in a blinded manner via a semiquantitative scheme according to a previously published method [17]: 0 = no peribronchial/perivascular infiltrates; 1 = scattered cells; 2 = moderate, unevenly distributed cells; 3 = abundant cells without clustering; and 4 = abundant cells that formed aggregates.

### 2.4. Serum IgG Isolation, N-Glycan Release, Purification and Detection

Serum was aliquoted and stored at −80 °C until analysis. For IgG purification, 20 μL of serum was diluted with 40 μL of 1× PBS and incubated with Protein G beads (Bestchrom, #04054715, Shanghai, China) with gentle rotation for 60 min at room temperature. Beads were washed three times with 1× PBS, and IgG was eluted with 200 μL of 100 mM glycine-HCl (pH 2.5). The eluate was immediately neutralized with 20 μL of 1 M ammonium bicarbonate (pH 9.0). IgG concentration was determined using the BCA Protein Assay Kit (Thermo, Pierce BCA Protein Assay Kit #23225, Waltham, MA, USA) to confirm successful extraction.

For N-glycan release, dried IgG samples were denatured with 10 μL of 2% SDS at 95 °C for 10 min, then cooled to room temperature. The reaction mixture was supplemented with 5 μL of 4% NP-40, 5 μL of 5× PBS, 1 μL of peptide-N-glycosidase F (PNGase F) (New England Biolabs, #P0704S, Ipswich, MA, USA), and 5 μL of water, and incubated at 37 °C overnight (16–18 h). Released N-glycans were labeled with 5 μL of 2-aminobenzamide (2-AB, Sigma-Aldrich, St. Louis, MO, USA) at 60 °C for 2 h. Glycans were purified using Sepharose CL-4B (Sigma-Aldrich; CL4B200) packed in a 96-well filter plate. Samples were eluted with water, dried by vacuum centrifugation, and reconstituted in 10 μL of water for UPLC analysis.

Labeled N-glycans were separated on a Nexera UPLC LC-30A system (Shimadzu, Kyoto, Japan) equipped with a fluorescence detector (excitation 330 nm, emission 420 nm). Separation was performed on a Waters BEH Glycan Amide column (150 × 2.1 mm, 1.7 μm, Waters, Milford, MA, USA) at 70 °C. Solvent A consisted of 100 mM ammonium formate (pH 4.4), and solvent B was acetonitrile. The linear gradient was 78–56% ACN (*v*/*v*) at a flow rate of 0.4 mL/min, column temperature = 60 °C, and injection volume 2 μL.

### 2.5. Statistical Analysis

Twelve individual glycan peaks (GP1-GP12) were quantified as relative abundances by normalizing individual peak intensities to the total sum of all 12 glycan intensities, the proposed structures were according to previous articles [18,19], in which these IgG N-glycan structures were confirmed by mass spectrometry, supporting the reliability of the current glycomic analysis. Five derived glycan traits (galactosylation, sialylation, monogalactosylation digalactosylation, and bisecting glycans) were calculated from the relative abundances of individual glycans according to their type. For two compared groups, Student’s *t*-test was used to evaluate statistical significance; *p*-value < 0.05 was considered statistically significant (*: *p* < 0.05; **: *p* < 0.01; ***: *p* < 0.001). Benjamini–Hochberg FDR correction was applied for multiple comparisons, with adjusted *p* values reported in Supplementary Table S1. The lung histopathology score was analyzed with the Mann–Whitney U test. All statistical analyses were performed using SPSS Statistics version 26.0 and GraphPad Prism version 9.0.

## 3. Results

### 3.1. Maternal Low-Dose LPS Exposure Shows a Trend Towards Attenuation of Offspring HDM-Induced Lung Inflammation

**Figure 1:**
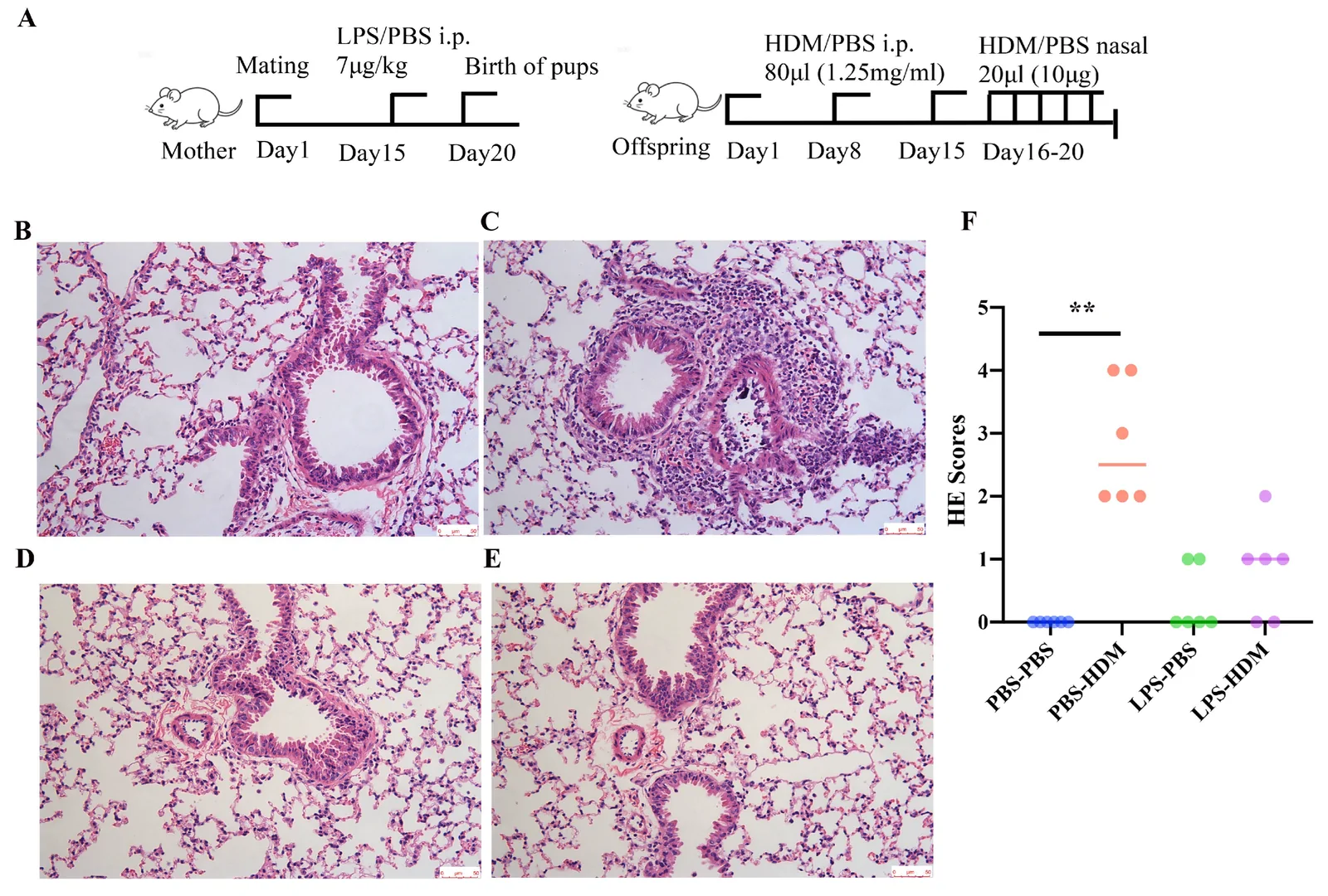
Maternal low-dose LPS exposure and HDM challenge in offspring mice. (**A**) Experimental timeline. Pregnant C57BL/6J mice received intraperitoneal LPS (7 µg/kg) or PBS on gestational day 15. Offspring were sensitized with HDM extract (80 µL, 1.25 mg/mL, intraperitoneal) on days 1, 8, and 15, followed by intranasal HDM (20 µL, 10 µg) on days 16–20. PBS-treated controls received equivalent volumes of PBS. (**B**–**E**) Representative HE-stained lung tissue sections (20× magnification) from the PBS-PBS, PBS-HDM, LPS-PBS, and LPS-HDM groups, respectively. (**F**) Lung inflammation scores. Data are shown as individual values with mean. Statistical analysis was performed using the Mann–Whitney U test, **: *p* < 0.01.

In this study, HE analysis (Figure 1B,C,F) showed that offspring in the PBS-HDM group had significantly greater inflammatory cell infiltration and airway remodeling in lung tissue compared with the PBS-PBS control group, confirming successful establishment of the HDM-induced allergic airway inflammation model. Compared with the PBS-HDM group, offspring in the LPS-HDM group exhibited lower lung tissue pathological damage scores (Figure 1D–F), showing a trend toward reduced allergic airway inflammation in offspring following maternal low-dose LPS exposure (Figure 1).

### 3.2. HDM Challenge Reduces IgG Galactosylation and Sialylation in Offspring

**Figure 2:**
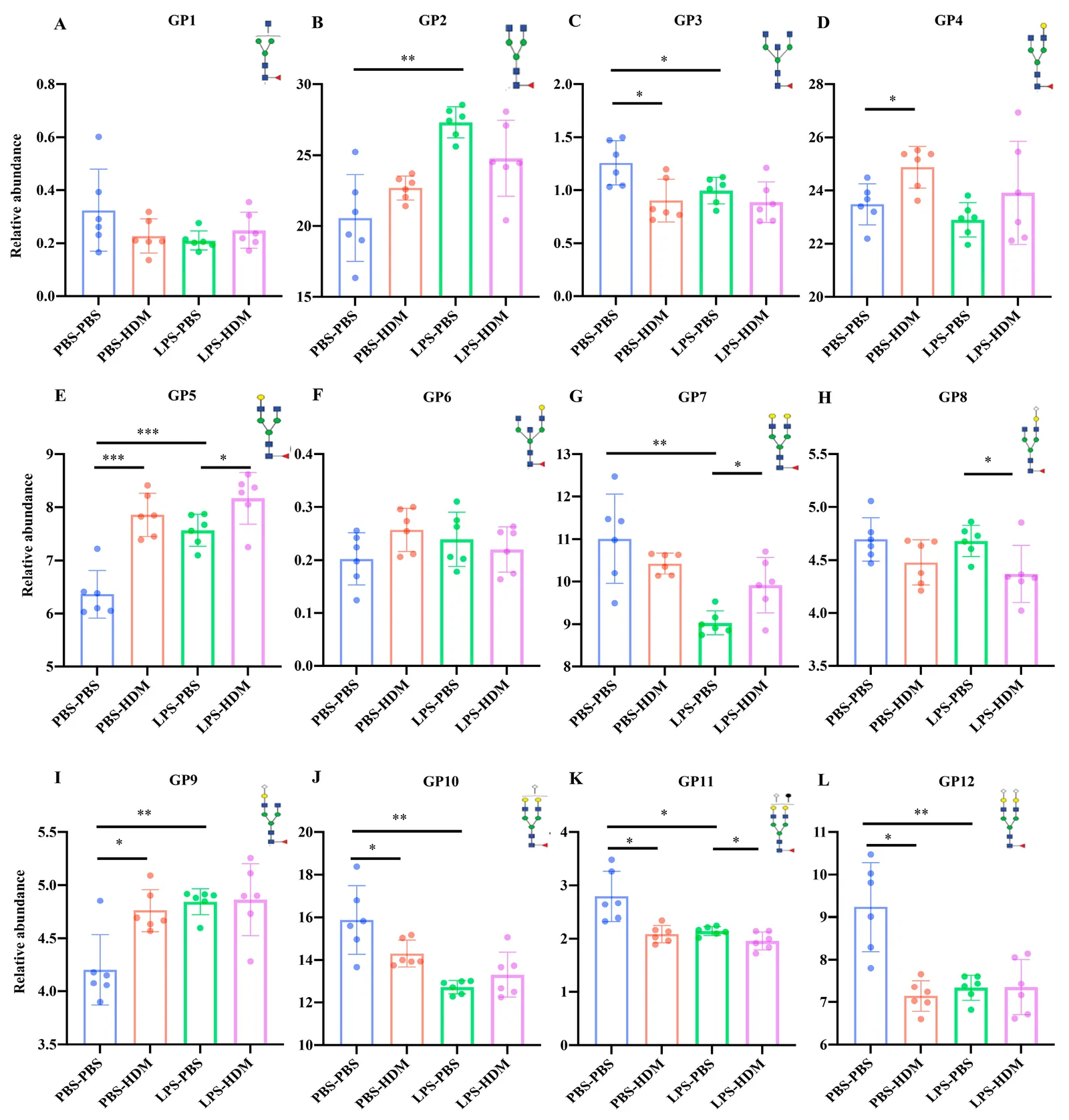
Relative abundance of individual IgG N-glycan peaks (GP1–GP12) in offspring serum. (**A**) GP1. (**B**) GP2. (**C**) GP3. (**D**) GP4. (**E**) GP5. (**F**) GP6. (**G**) GP7. (**H**) GP8. (**I**) GP9. (**J**) GP10. (**K**) GP11. (**L**) GP12. Serum IgG was purified by Protein G affinity chromatography, and N-glycans were released with PNGase F, labeled with 2-aminobenzamide, and analyzed by UPLC with fluorescence detection. Twelve glycan peaks were quantified as relative abundances. Data are shown as individual values with mean ± SD. Blue, PBS-PBS group; red, PBS-HDM group; green, LPS-PBS group; purple, LPS-HDM group. *t*-test *: *p* < 0.05; **: *p* < 0.01; ***: *p* < 0.001. Red triangle: fucose; blue square, N-acetylglucosamine; green circle, mannose; yellow circle, galactose (beta1–4 linked); black circle, galactose (alpha1–3linked); white diamond, N-glycolylneuraminic acid; purple diamond, N-acetylneuraminic acid.

**Figure 3:**
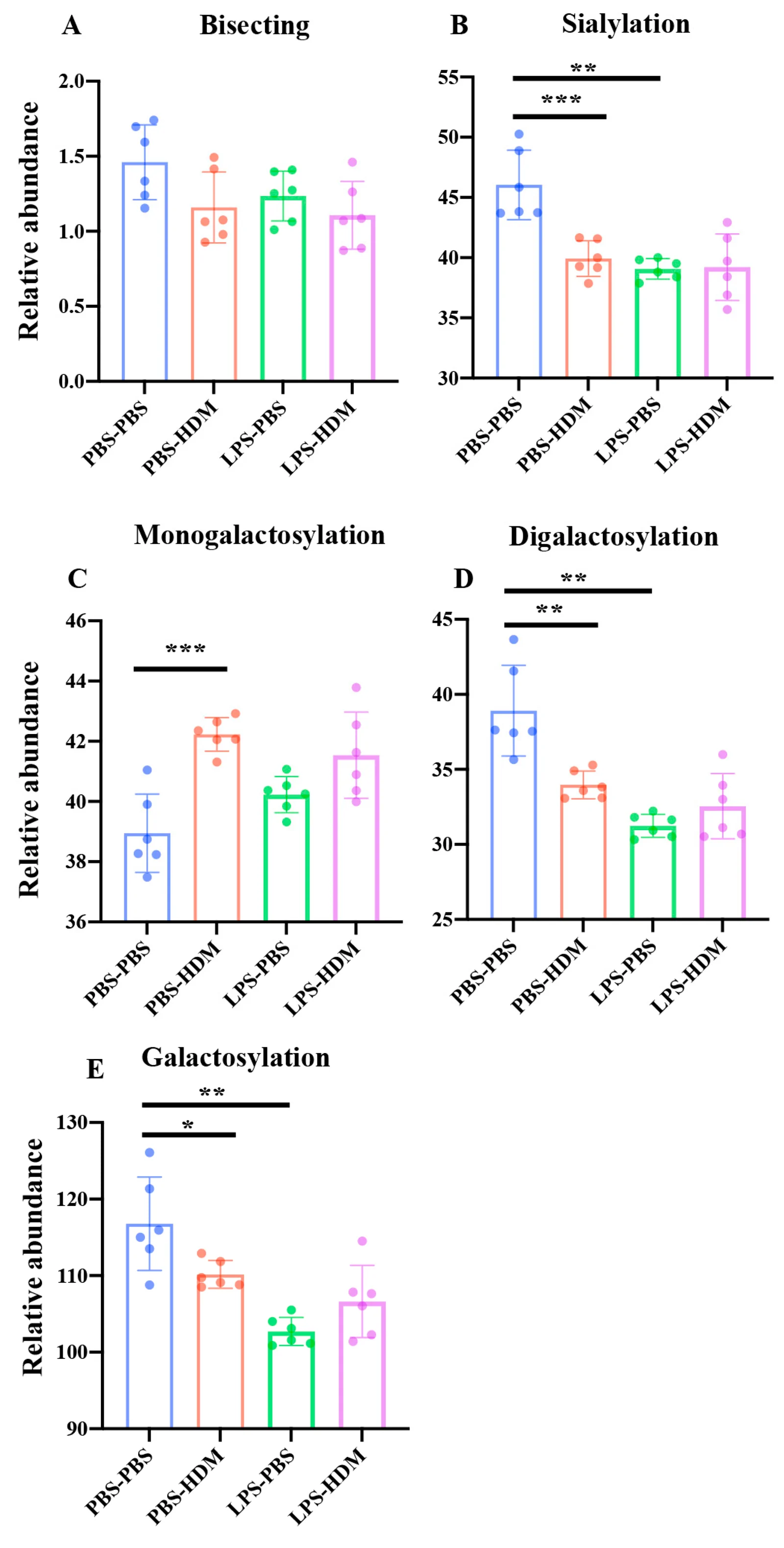
Derived glycan traits in offspring serum IgG. Derived glycan traits were calculated from the relative abundances of individual glycans. (**A**) Bisecting glycans. (**B**) Sialylation. (**C**) Monogalactosylation. (**D**) Digalactosylation. (**E**) Total galactosylation. Data are shown as individual values with mean ± SD. Blue: PBS-PBS group; red: PBS-HDM group; green: LPS-PBS group; purple: LPS-HDM group. *t*-test *: *p* < 0.05; **: *p* < 0.01; ***: *p* < 0.001.

We compared IgG glycan profiles between the PBS-PBS and PBS-HDM groups. GP4, GP5, and GP9 were significantly increased in the HDM group, whereas GP3, GP11, and GP12 were significantly decreased (Figure 2, Supplementary Table S1). Derived glycan trait analysis showed increased monogalactosylation and decreased digalactosylation, total galactosylation, and sialylation in the HDM group (Figure 3, Supplementary Table S1). Notably, total galactosylation did not survive Benjamini–Hochberg FDR correction. These results indicate that HDM-induced allergic airway inflammation is accompanied by reduced IgG galactosylation and sialylation in offspring. These findings suggest that low galactosylation (mainly digalactosylation, not monogalactosylation) and low sialylation may represent a common glycomic signature of inflammatory states.

### 3.3. Maternal Low-Dose LPS Exposure Alone Alters Offspring IgG Glycosylation but Does Not Rescue HDM-Induced Glycomic Changes

To investigate the effect of maternal low-dose LPS exposure alone on offspring IgG glycosylation, we compared the LPS-PBS and PBS-PBS groups. GP3, GP7, GP10, GP11, and GP12 were significantly decreased in the LPS-PBS group, whereas GP2, GP5, and GP9 were significantly increased (Figure 2, Supplementary Table S1). Analysis of derived glycan traits further showed that digalactosylation, total galactosylation, and sialylation were all significantly lower in the LPS-PBS group than in the PBS-PBS group (Figure 3, Supplementary Table S1). Low IgG galactosylation and sialylation suggest that maternal LPS exposure may induce a degree of inflammation or immune activation in offspring. Although maternal LPS pretreatment showed a trend toward reduced lung inflammation, it did not significantly alter IgG glycosylation profiles or reverse the HDM-induced reduction in IgG galactosylation and sialylation, when we compared the PBS-HDM and LPS-HDM groups directly. This indicates that, under HDM sensitization, maternal LPS pretreatment did not significantly reverse or alter the HDM-induced IgG glycomic remodeling.

## 4. Discussion

IgG sialylation is characteristically reduced in a range of pathological conditions, including chronic inflammatory diseases, malignancy, and aging [13]. In this study, offspring mice with HDM-induced allergic airway inflammation showed significantly decreased total IgG galactosylation and sialylation, which closely matches the inflammation-associated low-galactosylation (mainly digalactosylation) and sialylation phenotype reported previously [20,21,22]. To assess the robustness of this finding, we performed parallel validation in an ovalbumin (OVA)-induced asthma model (*n* = 4), and IgG galactosylation was similarly reduced (Supplementary Figure S1), further supporting an association between allergic airway inflammation and IgG glycomic remodeling. Of note, clinical studies have shown that serum IgG (4) galactosylation is lower in asthma patients than in healthy controls [23], and that galactosylation and sialylation partially recover after allergen-specific immunotherapy and suppress inflammation [24]. These observations suggest that IgG galactosylation and sialylation may not simply be a passive reflection of inflammatory status, but may also contribute to asthma pathogenesis and treatment response.

Interestingly, maternal low-dose LPS exposure alone (LPS-PBS group) also caused reduced IgG galactosylation and sialylation in offspring, suggesting that LPS may activate the maternal or fetal immune system and induce an inflammation-like glycomic phenotype in offspring. However, a paradox emerges: since maternal LPS pretreatment produces a trend towards alleviation of HDM-induced allergic airway inflammation in offspring, one might expect inflammation improvement to be accompanied by recovery of IgG galactosylation and sialylation, yet no significant difference in IgG galactosylation and sialylation profiles was observed between the LPS-HDM and PBS-HDM groups.

We speculate that this glycosylation–inflammation decoupling may arise from the following mechanism. Maternal LPS exposure activates a T helper (Th)1-type immune response (for example, through induction of interferon-gamma), creating a Th1-biased immunological microenvironment in offspring that antagonizes HDM-induced Th2-type allergic responses [6]. However, Th1-type immune activation itself can also drive the low-IgG-galactosylation phenotype (Figure 3E). Therefore, in the LPS-HDM group, Th1 immune bias and Th2 allergic responses may reach a form of immune equilibrium, resulting in reduced overall inflammatory burden while IgG glycosylation remains in a low-galactosylation state. IgG galactosylation and sialylation change in various inflammatory responses and may participate in immune regulation. One additional speculative consideration is that allergic airway inflammation involves separable local pulmonary and systemic immune regulation, which could further contribute to the decoupling between lung inflammatory phenotype and circulating IgG glycan profiles. However, when multiple inflammatory responses—such as Th1 and Th2—coexist, the low-galactosylation and low-sialylation state of IgG glycosylation may exert additive rather than neutralizing effects. To test this hypothesis, future studies should systematically compare the differential effects of LPS alone, HDM alone, and combined LPS plus HDM treatment on Th1/Th2 cytokine profiles and IgG glycosylation; splenic and pulmonary Th1/Th2/Th17/regulatory T (Treg) cell subset distributions by flow cytometry; and mRNA and protein expression of glycosyltransferases (such as β-1,4-galactosyltransferase and sialyltransferase) to define the enzymatic basis of glycosylation changes.

Currently, research on the regulatory mechanisms of IgG N-glycosylation in maternal–fetal immune transfer remains limited. Although existing studies have confirmed that IgG glycosylation affects placental transport efficiency and neonatal Fc receptor (FcRn)-mediated trans-epithelial transfer [14], systematic investigation into how maternal immune stimuli (such as LPS exposure) reshape IgG glycoforms and thereby regulate offspring immune development is still lacking. Future work should include detection and comparison of serum IgG N-glycosylation profiles between LPS-treated and PBS control dams, to establish whether maternal LPS exposure directly remodels maternal IgG glycan structures; if maternal IgG glycosylation changes are confirmed, with further tracking of their persistence and functional status in offspring circulation, to distinguish between direct transfer of modified IgG and indirect immune programming via maternal cytokines or metabolites; and in vitro Fcγ receptor binding assays and complement activation experiments to assess functional differences between glycoforms, and to clarify whether glycosylation modifications contribute to offspring immune protection through regulation of antibody effector functions. These studies will provide critical evidence for understanding the transgenerational regulatory axis of LPS exposure, maternal immunity, and offspring glycoimmunity.

This study has several limitations. The sample size in each experimental group was relatively small. Larger sample sizes and subsequent validation in human cohorts are needed to generate findings with greater clinical relevance. We only employed male offspring; given well-documented sex differences in allergic susceptibility and IgG glycosylation patterns, future studies including female animals are necessary to assess the generalizability of our results. Furthermore, we did not quantify local pulmonary immune cell infiltration or circulating inflammatory cytokines, restricting our capacity to dissect causal immune pathways. Multiple mechanistic interpretations, including the Th1/Th2 equilibrium hypothesis and the potential dissociation between local airway inflammation and systemic IgG glycan remodeling, should therefore be considered preliminary and require independent experimental validation. This is the first study to examine maternal LPS effects on offspring IgG N-glycosylation in allergic airway inflammation. The finding that LPS produced a trend toward attenuation without restoring IgG galactosylation and sialylation indicates that the effects of prenatal LPS exposure and postnatal allergic sensitization on IgG glycosylation are not mutually antagonistic, but rather may be additive or convergent. This provides a basis for investigating how prenatal immune stimuli shape offspring antibody glycosylation and whether persistent glycomic signatures have functional consequences for immune protection or disease susceptibility.

## 5. Conclusions

This study demonstrates that both maternal low-dose LPS exposure and offspring HDM challenge decrease serum IgG galactosylation and sialylation in mice independently. Importantly, when both stimuli were present, IgG galactosylation and sialylation remained low, and they did not recover despite the trend towards attenuation of HDM-induced lung inflammation. This indicates that the effects of prenatal LPS exposure and postnatal allergic sensitization on IgG glycosylation are not mutually antagonistic, but rather may be additive or convergent. These findings provide the first evidence linking prenatal immune stimulation to transgenerational IgG glycomic changes, and suggest that persistent low galactosylation and sialylation may serve as a stable biomarker of immune priming in offspring.

## Data Availability

All data generated in the production of this manuscript will be freely available upon request. The original contributions presented in this study are included in the article. Further inquiries can be directed to the corresponding authors.

## Supplementary Materials

The following supporting information can be downloaded at https://www.mdpi.com/article/10.3390/biomedicines14091942/s1. Figure S1: Derived N-glycan traits of serum IgG in OVA and PBS mice; Table S1: Relative abundances of individual IgG N-glycan peaks and derived glycan traits in offspring serum.

## Abbreviations

The following abbreviations are used in this manuscript:

LPS	lipopolysaccharide
HDM	house dust mite
IgG	immunoglobulin G
PBS	phosphate-buffered saline
PNGase F	peptide-N-glycosidase F
2-AB	2-aminobenzamide
UPLC	ultra-performance liquid chromatography
HE	hematoxylin–eosin
DOHaD	developmental origins of health and disease
OVA	ovalbumin
FcRn	neonatal Fc receptor
Th	T helper
Treg	regulatory T
ADCC	antibody-dependent cellular cytotoxicity
CDC	complement-dependent cytotoxicity
