# Supplementary material for: Maternal Low-Dose Lipopolysaccharide Exposure and House Dust Mite Challenge Alter Serum IgG N-Glycosylation in Offspring Mice
Source: Biomedicines. 2026 Aug 29;14(9):1942. doi: 10.3390/biomedicines14091942 (PMC13604380; doi:10.3390/biomedicines14091942)
Supplement: Supplementary file 1 [file biomedicines-14-01942-s001.zip › Supplementary figure.pdf]

Supplemental Materials and Methods:

1. Ovalbumin (OVA)-induced allergic airway inflammation mouse model

A total of 8 male mice were randomly divided into two groups (n = 4 per group): OVA group and PBS control group. Mice in the OVA group were intraperitoneally sensitized with 0.1 mL of 0.01% OVA/$Al(OH)_3$ mixture (containing 10 μg OVA(Sigma, #A5503) and 2 mg $Al(OH)_3$ gel (Thermo Fisher Scientific, Imject Alum, #77161)) on day 1 and day 13. From day 25 to day 31, mice received daily aerosol-challenge with 1% OVA in PBS for 30 min per day. Mice in the PBS control group were subjected to identical procedural schedules, but received equivalent-volume PBS instead of OVA-containing reagents for both intraperitoneal injection and aerosol nebulization.

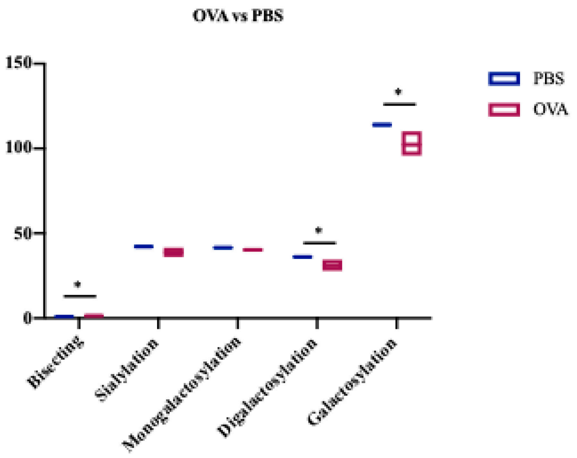


**Supplementary Figure S1. Derived N-glycan traits of serum IgG in OVA and PBS mice.** OVA-sensitised (red) and PBS control (blue), n= 4 per group.
